# The Synthesis of Narrowly Dispersed Poly(ε-caprolactone) Microspheres by Dispersion Polymerization Using a Homopolymer Poly(dodecyl acrylate) as the Stabilizer

**DOI:** 10.3390/polym16131911

**Published:** 2024-07-04

**Authors:** Yiling Wang, Chuangbang Xu, Qi Liu, Cuicui Guo, Shengmiao Zhang

**Affiliations:** School of Materials Science and Engineering, East China University of Science and Technology, Shanghai 200237, China; y30210864@mail.ecust.edu.cn (Y.W.); Y12233119@mail.ecust.edu.cn (C.X.); y82220331@mail.ecust.edu.cn (Q.L.); gwo3580@163.com (C.G.)

**Keywords:** dispersion polymerization, poly(dodecyl acrylate), poly(ε-caprolactone) microspheres

## Abstract

Using dodecyl acrylate as a raw material and 2-Cyanoprop-2-yl-dithiobenzoate as a chain transfer agent, poly(dodecyl acrylate) is synthesized by reversible addition–fragmentation chain transfer (RAFT) polymerization. Using poly(dodecyl acrylate) as stabilizers, narrowly dispersed poly(ε-caprolactone) microspheres with particle sizes ranging from 0.5 to 1.5 μm are successfully synthesized by ring-opening dispersion polymerization. The effects of the molecular weight of poly(dodecyl acrylate), the volume proportion of mixed solvent (i.e., 1,4-dioxane/heptane), and the reaction temperature on the particle size and its distribution are investigated. With careful control of the synthesis condition, microspheres can be obtained with a particle size distribution of 1.09 (*D*_w_/*D*_n_). The average particle size of poly(ε-caprolactone) microspheres decreased with the increase in the molecular weight of poly(dodecyl acrylate) and increased with the increase in the relative content of 1,4-dioxane. The uniformity of microspheres decreased with the increase in the polymerization temperature.

## 1. Introduction

Microspheres based on biocompatible materials have been widely used in biomedical fields, such as drug delivery and targeted therapies [1]. Among them, poly(ε-caprolactone) (PCL) microspheres have attracted increasing attention due to their prominent biodegradability and biocompatibility [2,3]. Microspheres with a uniform particle size regulate therapeutic efficacy, bioactivity, the drug release rate, and other important factors of microencapsulated drugs; thus, the preparation of microspheres with a controllable size is the key to determine the performance of a product [4]. So far, the main methods for the preparation of PCL microspheres include solvent evaporation [5], electrospraying [6], and membrane emulsion [7], which have the disadvantages of a cumbersome preparation process and a wide particle size distribution, causing commercial PCL microspheres with a uniform size to have a high cost [8].

The preparation of PCL microspheres by the polymerization of monomer CL can effectively avoid these drawbacks. For example, non-aqueous emulsion polymerization has been successfully applied in the preparation of uniform nanoscale PCL particles [9]. However, a method for preparing PCL microspheres with a micron scale size by the polymerization of CL is yet to be developed. Dispersion polymerization is considered a method for easily preparing polymer microspheres with a narrow particle distribution on a large scale. The particle size of the polymer microspheres obtained from dispersion polymerization commonly ranges from 0.1 to 15 μm [10,11,12,13]. PCL microspheres with a narrow particle size distribution can be efficiently prepared by ring-opening polymerization combining disperse polymerization with a cyclic ester monomer. Muranaka et al. [14] designed a poly(methyl dodecyl acrylate-*co*-hydroxyethyl methacrylate) (P(DMA-*co*-HEMA)) copolymer stabilizer with hydroxyl groups at the end group. With the hydroxyl group as the initiator, the ring-opening polymerization of lactide is carried out by dispersion polymerization in a xylene/heptane mixture (1:2, *v/v*) to form a PLA microsphere with a narrow particle size distribution. Huda et al. [15] prepared poly(n-octadecyl methacrylate-*co*-2-hydroxyethyl methacrylate) (P(OMA-*co*-HEMA)) by radical random copolymerization, and it was then used as the stabilizer for dispersion polymerization to prepare PCL microspheres in a xylene/heptane mixture (1:2, *v/v*).

In attempt to prepare micron-scale PCL microspheres with a narrow particle size distribution, in this work, a homopolymer poly(dodecyl acrylate) (PDA) with a narrow molecular weight distribution was designed as a stabilizer for the preparation of PCL microspheres with a narrow particle size distribution by dispersion polymerization in a mixed solvent (1,4-dioxane/n-heptane). The PDA was synthesized by reversible addition–fragmentation chain transfer (RAFT) polymerization. The particle size of the PCL microspheres was tuned by changing the molecular weight of the PDA, 1,4-dioxane/n-heptane ratio, and polymerization temperature. It is worth mentioning that since PCL, a flexible polyester material, has poor physical and mechanical properties [16], in order to change this status quo, we introduced an independently synthesized crosslinking agent, Bis (ε-caprolactone-4-yl) (BCY), for PCL based on previous reports [17] to enhance the physical properties of the PCL microspheres.

## 2. Materials and Methods

### 2.1. Materials

ε-Caprolactone (CL, 98%), dodecyl acrylate (DA, 98%), and 4,4-dicyclohexanone (98%) were purchased from Shanghai Macklin Biochemical Technology Co., Ltd. (Shanghai, China). 2-Cyanoprop-2-yl-dithiobenzoate (CPDB, chain transfer agent (CTA), 97%) was obtained from Sigma-Aldrich Shanghai Trading Co., Ltd. (Shanghai, China). 1,4-Dioxane (99.5%), diethylaluminum ethoxide (25% w/w in hexane), heptane (99.0%), dichloromethane (98%), m-chloroperoxide benzoic acid (m-CPBA, 99%), and methanol (99.5%) were purchased from Shanghai Titan Technology Co., Ltd. (Shanghai, China). Toluene and 2,2′-azobis (isobutyronitrile) (AIBN, 99.7%) were purchased from Sinopharm Chemical Reagent Co., Ltd. (Shanghai, China). CL was purified by distilling under a vacuum after treating it with CaH2 for 12 h. DA was purified by passing it through basic chromatographic aluminum oxide and anhydrous magnesium sulfate to remove the inhibitor and dissolve water before use. CL, toluene, 1,4-dioxane, and heptane were stored over 4-Å molecular sieves before use. Lab-made distilled water was used. All other reagents were used as received.

### 2.2. RAFT Polymerization of PDA as Stabilizer

**Scheme 1:**
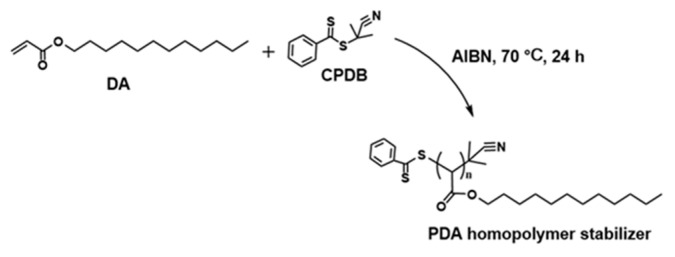
A schematic illustration of the synthesis of the PDA stabilizer.

PDA was synthesized according to Scheme 1. A series of PDA were synthesized by RAFT copolymerization with different molar ratios of monomer/chain transfer agent (DA/CPDB). In a typical copolymerization (PDA-2 in Table 1), DA (10 g, 42 mmol), CPDB (0.17 g, 0.8 mmol), AIBN (0.1 g, 0.6 mmol), and dehydrated toluene (34 g) were added to a three-necked flask. The solution was degassed by three freeze–pump–thaw cycles before it was heated at 70 °C for 24 h in N_2_. The solution was cooled down, concentrated by spin distillation, and then purified by precipitation with excess cold methanol. The product was dried in a vacuum oven at 40 °C for 24 h.

### 2.3. Synthesis of BCY

BCY was synthesized according to the literature with modifications [17]. The excess m-chloroperoxide benzoic acid (m-CPBA, 15 g, 87 mmol) was dissolved in 50 mL dichloromethane and placed in a flask. The flask was placed in an ice water bath equipped with a magnetic stirrer at 10 °C. After dissolving 4,4-dicyclohexanone (5 g, 25 mmol) in 50 mL dichloromethane, a constant-pressure funnel was used to drop the liquid into the flask within 1 h. The oxidation reaction lasted for 24 h. After the reaction, the excess m-CPBA powder was removed by suction filtration. The oil phase was washed successively with NaHCO_3_-saturated solution, NaHSO_3_-saturated solution, and water. After washing, the oil phase and water phase were separated, and the dichloromethane in the oil phase was evaporated to obtain a white powder. The white powder was then dried in a vacuum oven at 30 °C for 24 h to obtain the crosslinking agent, BCY.

### 2.4. Synthesis of PCL Microspheres

In a typical synthetic PCL microsphere experiment, CL (5.5 g, 48 mmol, containing 1 wt% crosslinker BCY) and PDA (0.22 g, 0.2% *w*/*v* in the solution) were added to 90 mL of 1,4-dioxane/heptane (1:8, *v/v*) to form a solution. The solution was transferred to a jacketed reactor equipped with mechanical stirring. The system was purged with nitrogen for 15 min and then cooled to 5 °C before diethylethoxyaluminum solution (25% *w*/*w* in hexane) at an amount of 0.52 mL was added to the reactor using a syringe. The reaction was carried out under a nitrogen atmosphere with mechanical stirring at 150 rpm for 10 h. PCL microspheres obtained from the solution by centrifugation were washed several times with excess heptane.

### 2.5. Characterization

^1^H-NMR spectra of PDA, BCY, and PCL were recorded using an AVANCE III 400 superconducting Fourier-transform nuclear magnetic resonance spectrometer (400 MHz, Bruker, Germany) in CDCl_3_. Due to its crosslinked structure, the PCL microspheres could not be completely dissolved in deuterated chloroform. Herein, ultrasonic heating was used to improve the solubility of the PCL microspheres in deuterated solvents [18]. Because of the low crosslinking degree, the PCL microspheres were swollen well in deuterated chloroform by ultrasonic heating method, and ^1^H-NMR spectra were obtained.

FT-IR spectra of PDA, BCY, and PCL microspheres were recorded using a Nicolet 6700 infrared spectrometer (Thermo Scientific, Waltham, MA, USA) with a wavelength ranging from 500 to 4000 cm^−1^. The molecular weights and the polydispersity index (PDI) of PDA and PCL were obtained by conventional GPC (Waters Corporation, Milford, MA, USA) with a system equipped with a Waters 1515 Isocratic HPLC pump, a S-3Waters 2414 refractive index detector, a Waters 2487 dual λ absorbance detector, and a set of Waters Styragel columns (HR3 (500–30,000), HR4 (5000–600,000), and HR5 (50,000–4,000,000), 7.8 × 300 mm, particle size: 5 μm). GPC measurements were carried out at 35 °C using THF as eluent with a flow rate of 1.0 mL/min. The system was calibrated with linear polystyrene standards.

The morphology of PCL microspheres was observed using a Hitachi S-4800 (Hitachi, Japan) field emission scanning electron microscope (FESEM) with an accelerating voltage of 10 kV. The average particle diameter (*D*_n_) and particle size distribution (*D*_w_/*D*_n_) were determined by SEM imaging using the following equations [19,20]:

(1) $$D_{n}=\frac{\sum_{i=0}^{n}D_{i}}{n}$$

(2) $$D_{w}=\frac{\sum_{i=0}^{n}D_{i}^{4}}{\sum_{i=0}^{n}D_{i}^{3}}$$

where *D_i_* is the diameter of the particle measured using Image J software, and *n* is the number of particles, which was above 100.

## 3. Results and Discussion

### 3.1. Synthesis of PDA

A stabilizer plays an important role in tuning the morphology of a polymer microsphere, including the particle size and its distribution in the dispersion polymerization [21]. Herein, PDA with a varied molecular weight was synthesized by RAFT polymerization to be used as the stabilizer for the disperse polymerization of CL (Table 1). As the molar ratio of DA to the chain transfer agent, CPDB, increased, the molecular weight of PDA increased, while the PDI (M_w_/M_n_) of the PDA was lower than 1.18, showing a narrow molecular weight distribution. This was considered a positive fact in the subsequent synthesis of polymer microspheres [20,22]. As the molecular weight distribution of the polymer stabilizer decreased, the particle size distribution (D_w_/D_n_) of the polymer microspheres decreased (Table 2).

**Figure 1:**
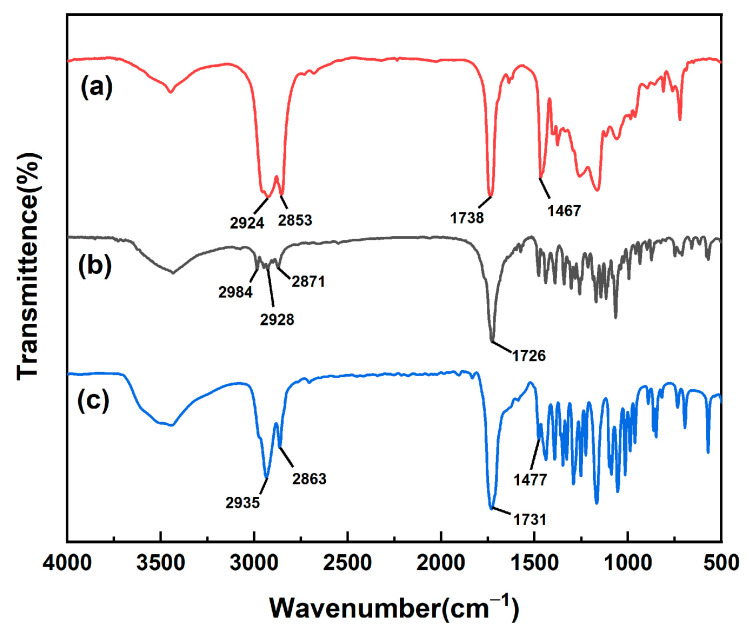
FT-IR spectra of (**a**) PDA stabilizer, (**b**) BCY crosslinker, and (**c**) PCL microsphere.

**Figure 2:**
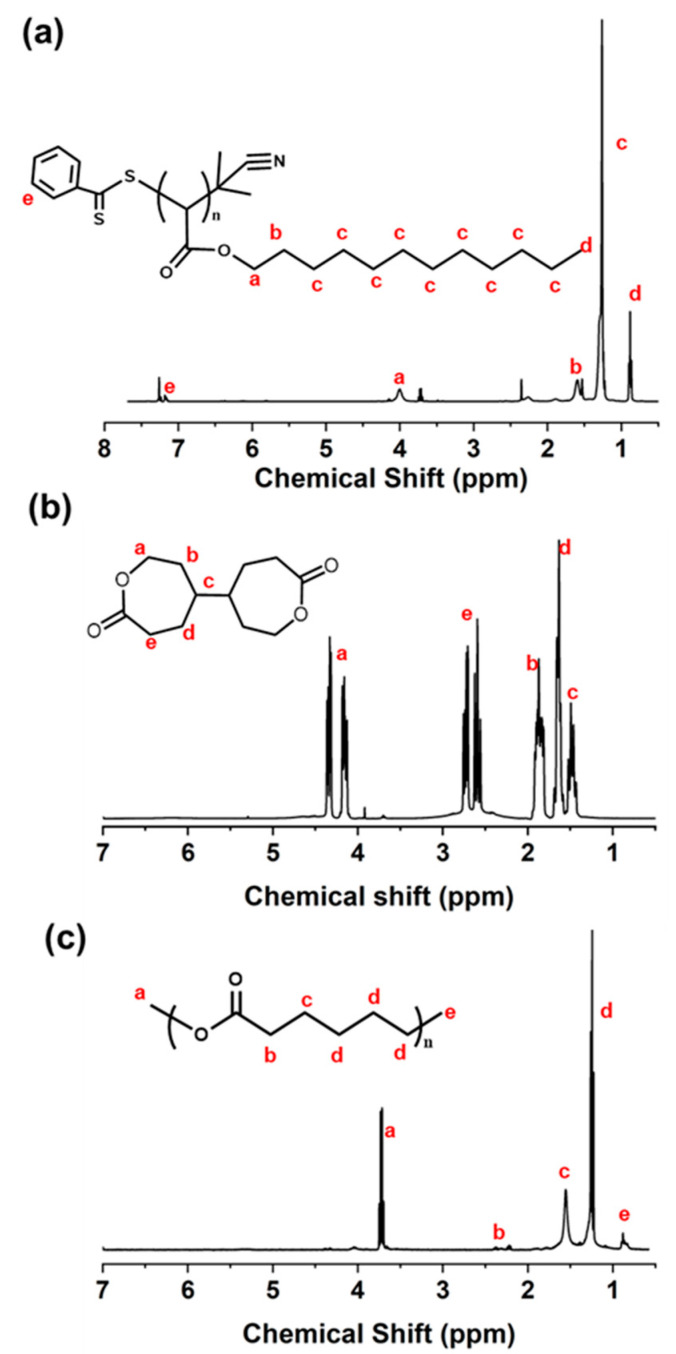
^1^H-NMR spectra of (**a**) PDA stabilizer, (**b**) BCY crosslinker, and (**c**) PCL microsphere.

The successful synthesis of PDA was verified by FT-IR. As shown in Figure 1a, the stretching vibration peak of C=C at 1800–1900 cm^−1^ was not found, proving that polymer polymerization was completed. Sharp C-H stretching vibration peaks at 2924 cm^−1^, 2853 cm^−1^, and 1467 cm^−1^ confirmed the long hydrophobic alkane chains of the polymer. Meanwhile, the strong peak at 1738 cm^−1^ belonged to the C=O stretching vibration peak, which confirmed the polyester structure of the PDA. The ^1^H-NMR spectrum of the PDA stabilizer is shown in Figure 2a. The peak at 4.0 ppm corresponded to the H of the -COOCH_2_- groups in PDA. The triple peak at 0.88 ppm corresponded to the hydrogen atom on the DA end methyl group. The presence of a strong peak at 1.26 ppm confirmed the long hydrophobic alkane chain of PDA. A weak peak at 7.16 ppm corresponded to the H in the benzene ring of CPDB. Thus, the structure of the PDA stabilizer was identified.

### 3.2. Synthesis of BCY

BCY, which has a dicyclocaprolactone structure, was synthesized to be the crosslinker to enhance the mechanical properties of the resulting PCL microspheres [23]. As shown in Figure 1b, there were weak peaks at about 2928 cm^−1^ and 2984 cm^−1^, which were methylene peaks on the bicyclic structure. A strong peak at 1726 cm^−1^ confirmed the presence of the carbonyl of BCY. BCY was further determined by ^1^H-NMR (Figure 2b); at the chemical shift of 4.33 ppm and 4.16 ppm, the two proton peaks represent the two hydrogens (t, 2H, -CH_2_-OOC-) on the methylene group attached to the ester group. Similarly, the proton peaks at 2.71 ppm and 2.59 ppm were hydrogen (t, 2H, -CH_2_-COO-) on the methylene group associated with the carbonyl group. Multiple peaks within the range of 1.81–1.92 ppm were substituted for two hydrogens (m, 2H, -CH_2_-CH_2_-OOC-) on the methylenes of the inter-ester site. The peak with a chemical shift of 1.63 ppm represented the methylene absorption peak of the carbonyl interposition (t, 2H, -CH_2_-CH_2_-COO-). The peak at 1.49 ppm represented the hydrogen atoms (q, 1H, -CH-CH_2_-) on the carbon where the two caprolactone rings join. Therefore, with the esteryl ortho and meta-H confirmed by ^1^H-NMR spectra, it could be concluded that 4,4′-dicyclohexanone successfully transformed into BCY with a dicyclohexanolactone structure.

### 3.3. Synthesis of PCL Microspheres

The PCL microspheres were synthesized by dispersion polymerization in 1,4-dioxane/heptane with PDA as a stabilizer, BCY as a crosslinker and diethylethoxy aluminum as an initiator. The synthesized PCL microspheres were analyzed by FT-IR (Figure 1c). The strong methylene stretching vibration peaks at 2935 cm^−1^ and 2863 cm^−1^ and the sharp C=O peak at 1731 cm^−1^ confirmed the long-chain polyester structure of PCL. Furthermore, the stretching vibration peak of -CH_3_ was detected at 1477 cm^−1^, indicating that the stabilizer PDA might be in the synthesized PCL microspheres. This conjecture was confirmed by the _1_H NMR spectrum (Figure 2c). The peaks at 3.70 ppm belonged to the CH_2_ adjacent to the carbonyl group. A weak peak of CH_3_ at 0.88 ppm belonged to the end group of PDA (also shown in Figure 2a), confirming the presence of PDA in the PCL microspheres. Moreover, by comparing Figure 2b,c, it was found that there were no characteristic peaks of the crosslinker BCY in the ^1^H-NMR spectrum of PCL microspheres, which proved that the crosslinker participated in the ring-opening polymerization of CL; thus, it was speculated that the crosslinking structure was successfully formed in the microspheres. In order to verify this conclusion, after freeze-drying, the PCL microspheres were placed in two different solvents, tetrahydrofuran and chloroform, and the solubility of the PCL microspheres in polar solvents was verified by ultrasonic heating and vibration. As a result, the PCL microspheres did not dissolve completely but remained in the solvent in the morphology of swelling, indicating that the PCL microspheres were crosslinked with a low crosslinking degree.

**Figure 3:**
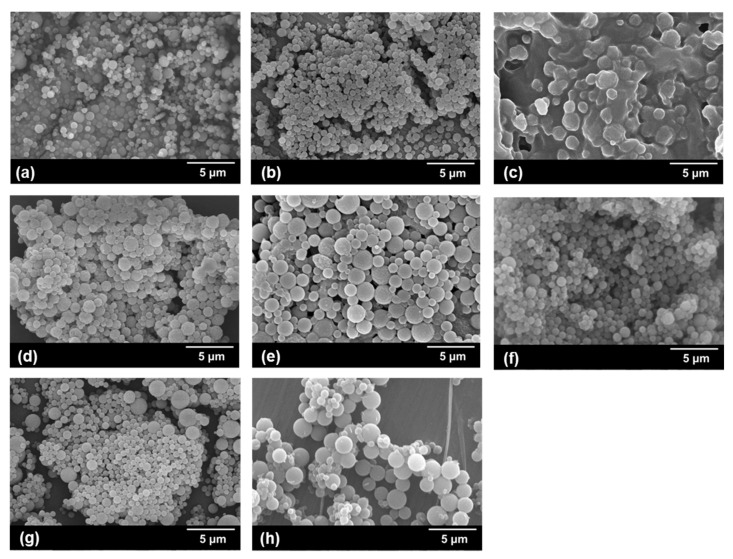
SEM images of PCL microspheres. (**a**) S1, (**b**) S2, (**c**) S3, (**d**) S4, (**e**) S5, (**f**) S6, (**g**) S7, and (**h**) S8.

**Figure 4:**
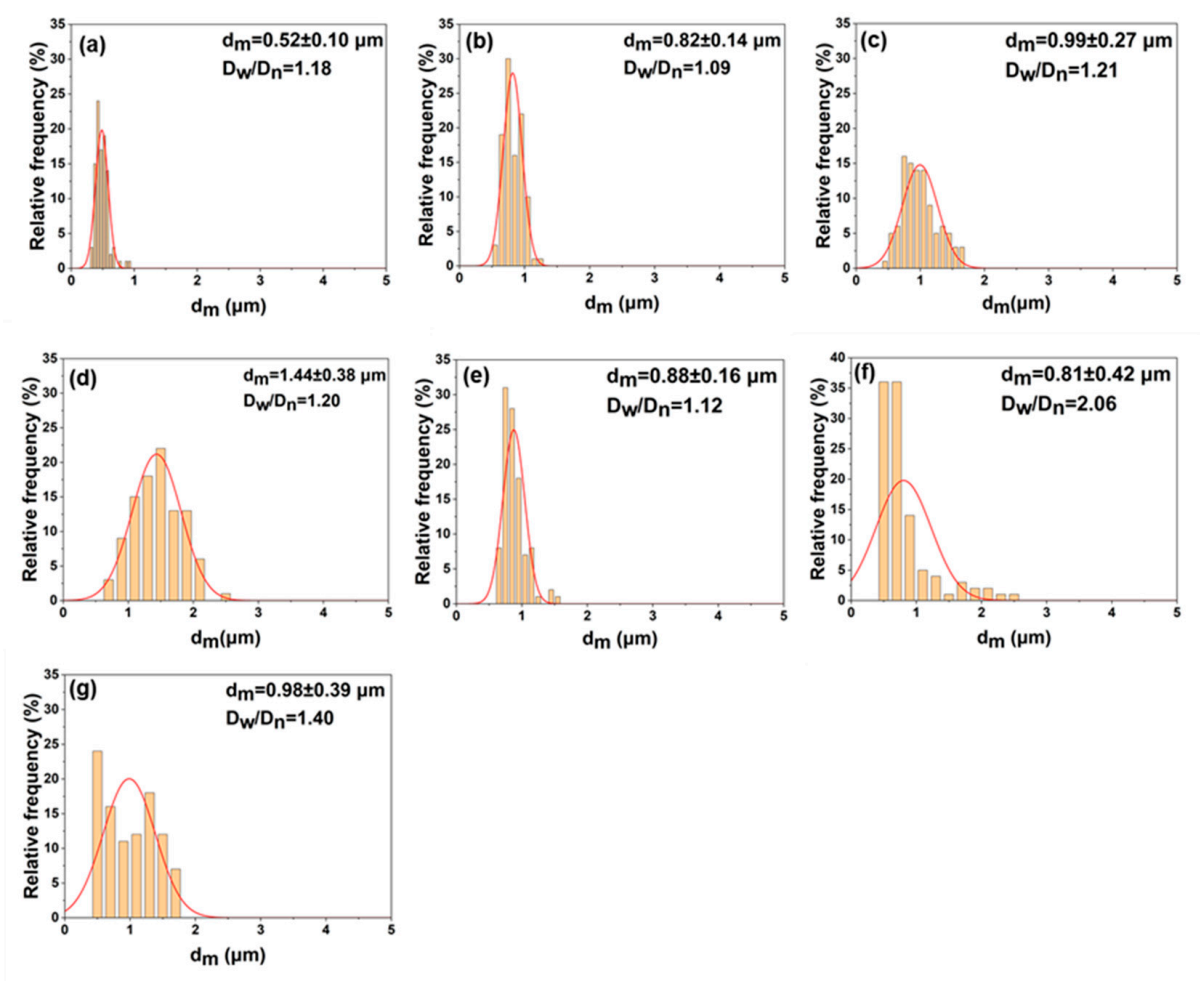
Size distributions of PCL microspheres from SEM images. (**a**) S1, (**b**) S2, (**c**) S4, (**d**) S5, (**e**) S6, (**f**) S7, and (**g**) S8.

As shown in Figure 3, a series of PCL microspheres were synthesized. The effects of the PDA molecular weight, volume ratio of the mixed solvent, and polymerization temperature on the morphologies of the PCL microspheres were investigated (Table 3). It was found that the PCL microspheres with a smooth surface and good spherical shape were obtained with the high-molecular-weight PDA as a stabilizer (M_n_ = 14,300 or 39,200 (Figure 3a,b). When the PCL microspheres were prepared using the PDA with a molecular weight of about 5000 as the stabilizer, the PCL particles were not spherical, with cohesive irregularly shaped blocks (Figure 3c), which could have been due to the fact that if the chain of the stabilizer is short, it cannot tangle and aggregate effectively on the surface of the primary particles, resulting in a final product of poor sphericity [24,25,26,27,28]. Therefore, it is necessary to use polymer stabilizers with larger molecular weights to prepare PCL microspheres with regular morphology. Meanwhile, as shown in Table 3 (S1 and S2) and Figure 4a,b, an increase in the molecular weight of the PDA decreased the particle size, indicating that the PDA with a larger molecular weight was a more effective stabilizer and therefore resulted a smaller particle size [29]. The D_w_/D_n_ of PDA-4-stabilized PCL microspheres prepared by free radical polymerization is significantly higher than that of S1 and S2 (Table 3 (S8) and Figure 4g), and the microsphere uniformity is worse, which confirms the advantages of a PDA stabilizer with a narrow molecular weight distribution prepared by RAFT polymerization in the process of dispersion polymerization of PCL microspheres: the narrower the molecular weight distribution of the macromolecular stabilizer used in dispersion polymerization, the higher the uniformity of the PCL microspheres prepared by dispersion polymerization.

The volume ratio of 1,4-dioxane/heptane (i.e., reaction media) and the polymerization temperature were also considered important factors impacting the tuning of the particle morphology in dispersion polymerization [30]. Herein, the influences of the volume ratio of 1,4-dioxane/heptane and the polymerization temperature on the particle size and distribution of the PCL microspheres were studied (Table 3, S2, S4–S7). With the increase in the volume ratio of 1,4-dioxane/heptane from 1/8 to 1.5/7.5, the particle size of the PCL microspheres increased from 0.82 to 1.44 μm. The oligomer precipitation mechanism believes that the oligomer formed in early reaction can be dissolved in a polymerization solvent [31]. Oligomers precipitate from the solvent when they reach a critical degree of polymerization, and the nucleus of a particle is formed [32]. In this system, increasing the ratio of 1,4-dioxane/heptane would reduce the precipitation nucleation rate; as a result, the particle size of the initial nucleus formed by the polymer in the nucleation stage of dispersion polymerization is larger, and microspheres with a larger particle size are eventually formed after chain growth. In addition, as shown in Figure 4b, the S2 prepared with the 1,4-dioxane/heptane ratio of 1/8 has the narrowest particle size distribution of 1.09.

Because the T_g_ of PCL is low [33], diethylethoxyaluminum, a very active covalent metal alkoxy compound, was chosen as a catalyst for the ring-opening polymerization of CL at a low temperature. The influence of the polymerization temperature (0–10 °C) on the particle size of PCL microspheres and its distribution was investigated (Table 3, S2, S6, and S7). It was found that increasing the polymerization temperature from 0 to 10 °C had a slight effect on the particle size; however, it significantly affected the uniformity of the microspheres. Varying the polymerization temperature in the range of 0 to 10 °C had little effect on the solubility of both CL and the produced PCL in solvents. Thus, the change in the polymerization temperature did not affect the nucleation rate or the particle size (Figure 3 and Figure 4). The particle size distribution of the PCL microspheres increased significantly (Table 3 and Figure 4) because there was an increase in the activity of initiator diethyl ethoxylaluminum caused by a rise in temperature [34], which led to a more intense polymerization reaction. In the process of dispersion polymerization, the growth stage of the particles after nucleation was greatly shortened, so the growth stage of the microspheres became rapid and unstable, and the resulting microspheres had an uneven particle size distribution.

## 4. Conclusions

Micron-scale PCL microspheres with a narrow size distribution were successfully prepared by ring-opening dispersion polymerization. A series of uniform PDA (*M*_w_/*M*_n_ ≤ 1.18) were synthesized by Raft polymerization, with the *M*_n_ value varying from 4600 to 39,200 as a stabilizer for dispersion polymerization. The PDA proved to be an efficient stabilizer for the dispersion polymerization of CL in a mixed solvent involving 1,4-dioxane/heptane. With the as-synthesized BCY as a crosslinker, the PCL microspheres had a good spherical morphology. The particle size of the PCL microspheres decreased with an increase in the PDA’s molecular weight and/or a decrease in the ratio of 1,4-dioxane/heptane. Changing the polymerization temperature did not change the particle size of the PCL microspheres, but it broadened their distribution. Using a 1,4-dioxane/heptane mixture (1/8, *v/v*) as a solvent and PDA (*M*_n_ = 14,300) as a stabilizer, sub-micron PCL microspheres with a narrow particle size distribution (*D*_w_/*D*_n_ = 1.09) could be prepared at 5 °C. Consequently, PCL microspheres with a narrow size distribution were obtained by using as-synthesized PDA as a stabilizer via dispersion polymerization in 1,4-dioxane/heptane. In general, this work developed a facile and easily scale-up method for the synthesis of micron-scale PCL microspheres with a narrow size distribution.

## Figures and Tables

**Table 1:** PDA homopolymers obtained with different molar ratios of DA/CPDB.

Sample	DA (mmol)	CPDB (mmol)	DA/CPDB (Molar Ratio)	*M*_n_ in Theory	*M*_n_ Determined by GPC	*M*_w_ Determined by GPC	*M*_w_/*M*_n_
PDA-1	42	0.3	140:1	33,800	39,200	45,900	1.17
PDA-2	42	0.8	53:1	13,000	14,300	17,000	1.18
PDA-3	42	2	21:1	5200	4600	5200	1.12
PDA-4	42	0	-	17,000	15,600	41,000	2.62

**Table 2:** PCL microspheres obtained with different PDA.

Sample	Stabilizer	*M*_n_ of PDA	*M*_w_/*M*_n_ of PDA	*D*_w_/*D*_n_ of PCL Microspheres
S1	PDA-1	39,200	1.17	1.18
S2	PDA-2	14,300	1.18	1.09
S8	PDA-4	15,600	2.62	1.40

**Table 3:** Synthesis of PCL microspheres.

Sample	Stabilizer	1,4-Dioxane/Heptane (*v/v*)	*T*^a^ (°C)	PCL Morphology	*D*_n_^b^ (μm)	*D*_w_/*D*_n_^c^
S1	PDA-1	1/8	5	Spherical	0.52	1.18
S2	PDA-2	1/8	5	Spherical	0.82	1.09
S3	PDA-3	1/8	5	Agglomerated *	-	-
S4	PDA-2	1.2/7.8	5	Spherical	0.99	1.21
S5	PDA-2	1.5/7.5	5	Spherical	1.44	1.20
S6	PDA-2	1/8	0	Spherical	0.88	1.12
S7	PDA-2	1/8	10	Spherical	0.81	2.06
S8	PDA-4	1/8	5	Spherical	0.98	1.40

^a^ The reaction temperature. ^b^ The average particle size of the PCL microspheres. ^c^ The particle size distribution. * The molecular weight of PDA-3 is too low to occupy enough area on the surfaces of the PCL microspheres, resulting in unstable and non-spherical PCL particles.

## Data Availability

The original contributions presented in the study are included in the article/supplementary material, further inquiries can be directed to the corresponding author/s.

## Supplementary Materials

The following supporting information can be downloaded at: https://www.mdpi.com/article/10.3390/polym16131911/s1, Figure S1: GPC curves of PDA homopolymer stabilizers with different molecular weights; Figure S2: Swelling verification of cross-linked PCL microspheres in polar solvents.
