# Supplementary material for: The Synthesis of Narrowly Dispersed Poly(ε-caprolactone) Microspheres by Dispersion Polymerization Using a Homopolymer Poly(dodecyl acrylate) as the Stabilizer
Source: Polymers (Basel). 2024 Jul 4;16(13):1911. doi: 10.3390/polym16131911 (PMC11243806; doi:10.3390/polym16131911)
Supplement: Supplementary file 1 [file polymers-16-01911-s001.zip › polymers-3037333-Supplementary.pdf]

Supporting information for

The Synthesis of Narrowly Dispersed Poly(ε-caprolactone) Microspheres by Dispersion Polymerization Using a Homopolymer Poly(dodecyl acrylate) as the Stabilizer

Yiling Wang, Chuangbang Xu, Qi Liu, Cuicui Guo and Shengmiao Zhang *

School of Materials Science and Engineering, East China University of Science and Technology, Shanghai 200237, China; y30210864@mail.ecust.edu.cn (Y.W.); Y12233119@mail.ecust.edu.cn (C.X.); y82220331@mail.ecust.edu.cn (Q.L.); gwo3580@163.com (C.G.)

* Correspondence: shmzhang@ecust.edu.cn

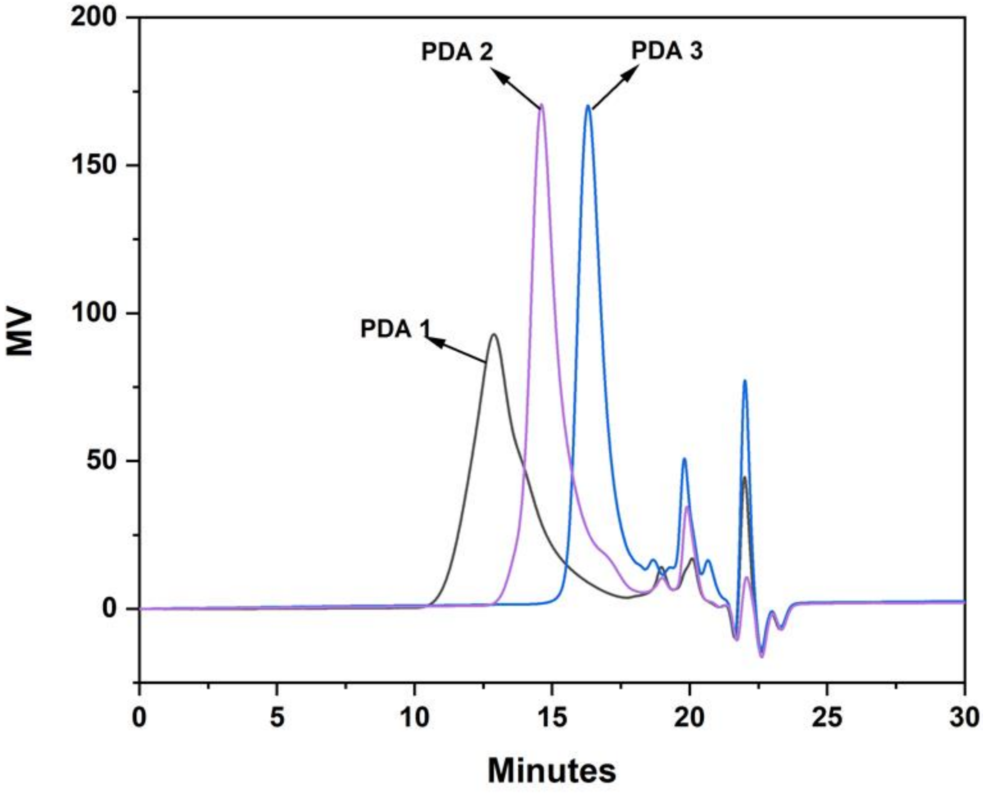

Figure S1. GPC curves of PDA homopolymer stabilizers with different molecular weights.

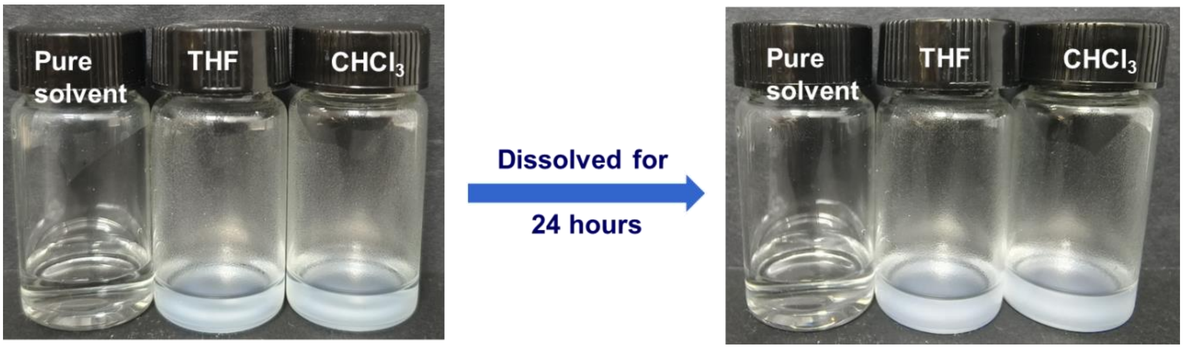

Figure S2. Swelling verification of cross-linked PCL microspheres in polar solvents.
